# Extracellular Vesicles as Drug Transporters

**DOI:** 10.3390/ijms241210267

**Published:** 2023-06-17

**Authors:** Monika Nowak, Julia Górczyńska, Katarzyna Kołodzińska, Jakub Rubin, Anna Choromańska

**Affiliations:** 1 Faculty of Medicine, Wroclaw Medical University, Mikulicza-Radeckiego 5, 50-345 Wroclaw, Poland; monika.nowak@student.umw.edu.pl (M.N.); julia.gorczynska@student.umw.edu.pl (J.G.); jakub.rubin@student.umw.edu.pl (J.R.); 2 Faculty of Biology and Animal Science, Wroclaw University of Environmental and Life Sciences, Norwida 25, 50-375 Wroclaw, Poland; kolodzinska.kasia@gmail.com; 3 Department of Molecular and Cellular Biology, Wroclaw Medical University, Borowska 211A, 50-556 Wroclaw, Poland

**Keywords:** extracellular vesicles, drug delivery, nanomedicine, engineering, cancer treatment

## Abstract

Extracellular vesicles (EVs) are lipid bilayer-delimited particles. According to their size and synthesis pathway, EVs can be classified into exosomes, ectosomes (microvesicles), and apoptotic bodies. Extracellular vesicles are of great interest to the scientific community due to their role in cell-to-cell communication and their drug-carrying abilities. The study aims to show opportunities for the application of EVs as drug transporters by considering techniques applicable for loading EVs, current limitations, and the uniqueness of this idea compared to other drug transporters. In addition, EVs have therapeutic potential in anticancer therapy (especially in glioblastoma, pancreatic cancer, and breast cancer).

## 1. Introduction

**Figure 1:**
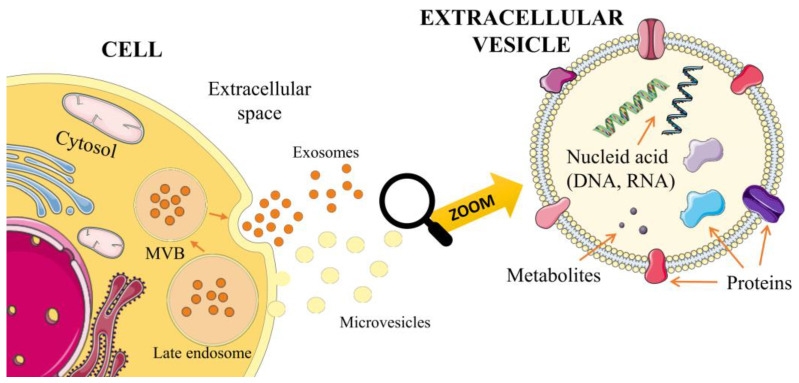
Biogenesis of exosomes and microvesicles.

Extracellular vesicles (EVs) are a heterogeneous population of cell-derived membrane particles, and are carriers for transferring substances, such as proteins, lipids, RNA, or DNA, at higher concentrations than in their environment (Figure 1).

Moreover, they can adhere to particular cells or tissues in a receptor-controlled manner to release their contents into the corresponding target structures. Therefore, they play an essential role in intercellular communication [1,2]. EVs are released from cell surfaces by normal, cancerous, and apoptotic cells [2,3]. They have also been found in many body fluids, including saliva, urine, milk, and amniotic fluid [1]. The current classification of EVs is based mainly on their size and the cellular compartment from which they come. EVs can be divided into three major categories: exosomes, ectosomes, and apoptotic bodies [4,5]. These are characterized in the table below (Table 1).

Exosomes are produced within multivesicular endosomes in the process of MVB maturation, in which the endosomal membrane buds inward. They are secreted as a result of MVB fusion with the cell membrane.

Ectosomes are believed to be formed by pinching off the plasma membrane as the cell contracts in unique spaces. This process is thought to be dependent on certain cytoskeleton components, i.e., microtubules and actin fibers with molecular motors and SNARE proteins.

Apoptotic bodies are formed during apoptosis as a result of blebbing, fragmenting, and dividing of the cell membrane [5].

EVs are signal carriers involved in the homeostasis of many bodily functions and participate in cell development, such as cell differentiation [12]. They are used for cell interactions, which can occur unilaterally or bilaterally. They can both deliver EV cargo and modulate target cells, for example, by activating immune cells [13]. Extracellular vesicles can fuse with target cells in various ways, including endocytosis, phagocytosis, or direct fusion with the plasma membrane. That process can occur through receptor–ligand interactions [14]. EVs induce profound phenotypic changes in the tumor microenvironment (TME) by transferring molecules, including oncoproteins and oncopeptides, from donor cells to recipient cells. Tumor cells secrete more EVs than non-malignant cells. Cancer cell growth, invasion, and metastasis depend on bidirectional cell-to-cell communication in complex tissue environments. A growing body of research has focused on assessing the role of EVs in tumor growth and metastasis [15]. The effect of exosome-derived miRNAs on angiogenesis necessary for cancerous tumor growth has also been pointed out. One example is miRNAs from exosomes released from CD105+ renal cancer cells [16]. The miRNA molecules contained in exosomes are often specific for a particular type of cancer, such as breast cancer with miR-21 and miR-1246 (exosomes from plasma) [17], and Squamous cell carcinoma of the esophagus with miR-21 (serum exosomes) [18]. Therefore, EVs are considered good diagnostic markers for detecting early cancers in a minimally invasive manner [3]. A growing body of evidence points to the potential of EVs as carriers for effective drug delivery, which carries excellent pharmacological prospects and is still being developed [13].

In order to bring EVs into clinical settings, they can be isolated by several methods, including the most common, namely ultracentrifugation. These methods can be divided into the following categories: density-based, size-based, affinity-based, exosome precipitation, and microfluidic-based isolation [10]. These are outlined in the table below (Table 2).

## 2. EVs as Transporters—Opportunities and Limitations

The EV group includes exosomes, ectosomes, and apoptotic bodies, which are naturally responsible for the transport and communication between cells. On this basis, EVs can perfectly function as drug transporters. However, recent years of research on the concept have made it clear that the idea has several limitations, which despite extensive research, still limit the clinical use of EVs.

The EVs have a diverse and heterogeneous structure consisting of proteins, lipids, and nucleic acids. Their size and characteristics vary depending on biogenesis pathways and mother cells [13]. They are present in almost all body fluids and cells, allowing them to differentiate and create targeted transport on an extensive scale [1,35]. Moreover, peptide ligand surface modification of the EVs can be successfully carried out using genetic engineering, resulting in the conjugation of additional fusion proteins [36]. It targets specific tissues and allows signaling pathways to inhibit or induce events [35]. The small size of the EVs (30 nm–5 μm) and their biocompatible, differentiated structure also creates the possibility of transporting drugs, enzymes, or nucleic acids across the blood–brain barrier [35,37,38]. This extraordinary feature makes the EVs of great importance, since most drugs cannot enter the brain.

In addition, the transport of substances through the EVs, for instance, dopamine, increases their distribution to the brain with minimized toxicity [38]. Furthermore, it has been shown that significantly lower immune clearance is observed during therapy with EVs than with artificial liposomes. This fact applies to the passage of nanocarriers into cells and cargo insertion [35,36]. The EVs characterized by excellent transcellular permeability [36]. Phagocytosis, macropinocytosis, lipid raft interactions, caveolae, receptor-mediated endocytosis, clathrin interactions, direct fusion, or binding are possible cellular pathways for the EVs to enter a cell in a variety of tissues [13,35]. Depending on the cargo, the possible loading methods can be passive, mechanical, or chemical. However, no universal procedure has been found to reproducibly charge the EVs with different cargos. In addition, the EVs’ lipid membrane structure facilitates the charging of many hydrophobic therapeutics [38,39]. Cell-free vaccines are developed based on bacterial EVs with antigens and virulence factors [40].

On the other hand, EVs have limited loading capacity due to different sizes, which poses problems with planning therapeutic applications [41]. Furthermore, various EV sizes cause difficulty in standardization and high-throughput production processes. Another area for improvement is the short half-life of the EVs, much faster than for liposomes, complicating the cargo delivery and accumulation in target tissues [42].

**Figure 2:**
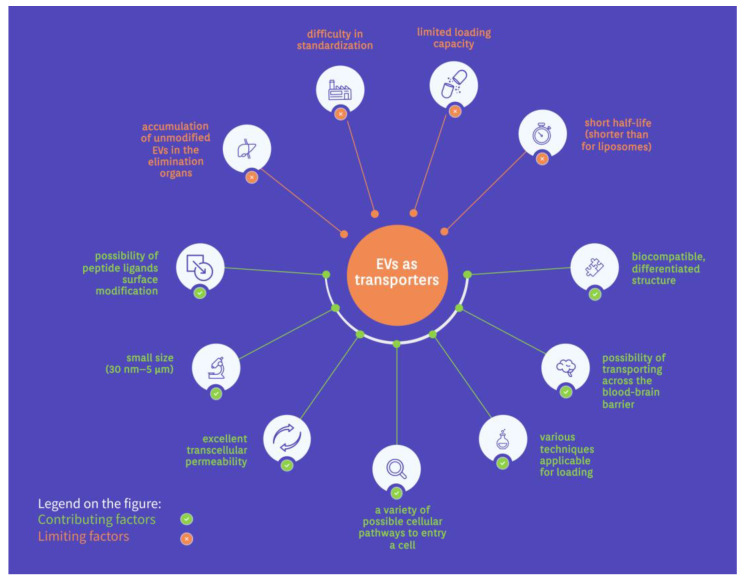
The opportunities and limitations of EVs as drug transporters.

Moreover, the rapid accumulation of unmodified EVs in the elimination organs (liver, spleen, lungs) is a highly limiting factor for drug distribution [13]. This problem has been observed for EVs regardless of their tissue origin. Transported cargo must also face possible endosomal escape, which is related to the acidic environment of the lysosomal pathway [13,36]. Differential expression of cell surface receptors in the recipient’s body is another challenge to overcome in designing EV drug therapies. Furthermore, EVs’ functional heterogeneity also affects cellular pathways, such as immunomodulation [35]. The lack of understanding of EVs is blamed on the difficulty of experimental observation, structural complexities, and the need for appropriate research methods [13]. However, despite all these problems, using EVs in drug therapy is promising. Therefore, it is extensively researched worldwide. The opportunities and limitations of EVs as drug transporters are illustrated in Figure 2.

## 3. Techniques Applicable for Loading EVs

**Figure 3:**
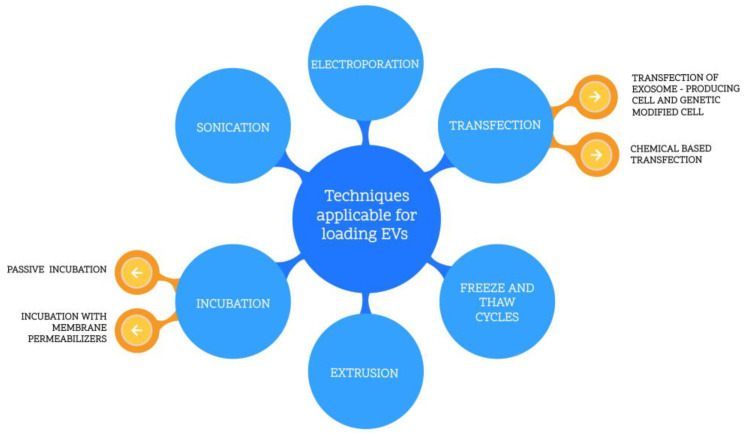
Techniques applicable for loading EVs.

There are several methods of introducing the drug into EVs, as listed in Figure 3.

### 3.1. Chemical Based Transfection

Chemical reactants can be successfully utilized to insert nucleic acids into the EVs by transfection. Zhang et al. [43] presented a modified method protocol for loading miRNAs into the EVs using calcium chloride. First, the miRNAs were mixed with exosomes in a PBS buffer. Subsequently, CaCl_2_ solution was gradually added until it reached a concentration of 0.1 M. Next, the sample was cooled in ice, then rapidly heated at 42 °C and cooled again in ice. The final step was to isolate the exosomes from the mixture. In this case, it was suspected that the formed CaCl_2_–RNA complex might be introduced into the exosome under heat shock conditions, resulting in membrane liquefaction. The authors compared the results of this method with electroporation, performed as a reference method [43]. In both cases, miRNAs were successfully loaded into the EVs with similar efficiency. However, the simplicity and the lack of necessary advanced equipment are in favor of the incubation method. In another paper [44], commercial reagent lipofectamine was used as a chemical transfection reactant for loading siRNA into the EVs. The siRNA was mixed with lipofectamine and incubated for 10 min at room temperature. Subsequently, exosome suspension was added and incubated for 30 min. In the end, the solution was filtered from the excess micelles. The transfection reagent HiPerFect was applied likewise to transfect double-stranded siRNA into exosomes. The siRNA was incubated in PBS solution with HiPerFect (QIAGEN, 19300 Germantown, MD 20874, USA) for 10 min at room temperature. Finally, the excess siRNA was purified using latex beads [45]. The following method is fast and uncomplicated. However, it requires additional purification processes and a specific reagent, which increases the cost.

### 3.2. Incubation (Permeabilized Membrane or Passive)

Incubation is the simplest method to load EVs. Two types of incubation can be distinguished: incubation utilizing a permeabilized membrane or passive incubation. Permeabilizers are often surfactants that form complexes or interact with the EVs’ membrane, resulting in pore formation and enhanced cargo loading [46]. Sun et al. [39] incubated curcumin with a solution of the EVs in PBS for 5 min at 22 °C, then centrifuged the solution in a sucrose gradient for 1.5 h at 36,000 rpm. The co-incubation method facilitated loading the EVs. Nevertheless, its efficiency is cargo-dependent. In this case, it was hydrophobic curcumin, which interacts with lipid membranes to allow the process. Various drug substances were loaded into the milk-derived exosomes: withaferin A, bilberry-derived anthocyanidins, curcumin, paclitaxel, and docetaxel [47]. Each was dissolved in ethanol, or a mixture of ethanol and acetonitrile, then added to the exosome solution and incubated at 22 °C. The samples were centrifuged for over two h to purify the loaded EVs. Ultimately the drug loading for the obtained exosomes varied from 10–40%. The value depended on the type and size of the drug. However, it was concluded that the solvent in a concentration of up to 10% did not affect the efficiency of the process [47]. In other studies [48], EVs, porphyrins, and saponins were incubated at room temperature for 10 min. It was concluded that the saponin-assisted method allows for an 11-fold increase in porphyrin loading compared to other passive incubation methods. The result was influenced by increased permeabilization caused by cholesterol complexes in the EVs’ membrane with saponins [46].

### 3.3. Extrusion

The extrusion method involves mixing a cargo solution with the EVs, then introducing the solution into an extruder as a syringe blocked with a porous membrane. It results in the membrane disruption and formation of the EVs with a size appropriate to the membrane used (usually 100–400 nm) [46]. Fuhrmann et al. [48] prepared EVs with porphyrin using a polycarbonate membrane with 400 nm pores in a syringe-based extruder at 42 °C. However, this procedure was repeated 31 times to obtain a single sample. Smaller-sized EVs were presented in Haney et al. [49], wherein authors applied a membrane with 200 nm pores for catalase loading. This extrusion was characterized by high efficiency. However, it caused numerous structural changes in EVs. In some cases, cytotoxicity is observed, in contrast to other methods of loading the same cargo into the EVs [48,49]. Fuhrmann et al. [48] observed the changing zeta potential for EVs received by the extrusion method.

### 3.4. Freeze and Thaw Cycles

The freeze and thaw cycles method typically consists of alternating cycles of temperature shocks: incubation at room temperature and freezing at −80 °C. The cycles are often repeated for enhanced loading efficiency. Haney et al. [49] applied the procedure to insert the catalase enzyme into macrophage-derived exosomes. The process consisted of three repeated cycles: 30 min of exosomes incubation at room temperature in PBS buffer with catalase, then rapid freezing of the samples at −80 °C, and re-thawing. The results were not satisfactory compared to other methods (sonication, extrusion, incubation). The loading efficiency obtained for freeze–thaw cycles was 14.7%, and the sonication and extrusion method results were more promising (26.1% and 22.2%, respectively). Nevertheless, incubation at room temperature yielded a much lower result of 4.7%. Furthermore, the catalytic activity of the catalase-loaded EVs prepared by different methods followed the same listed order. An analogous procedure was carried out to load the EVs with peptides composed of 34 amino acids [50]. Among the mentioned conditions, only the incubation time at room temperature was extended to 2 h. The method showed satisfactory results over various EV sizes (26 to 295 nm).

### 3.5. Electroporation

Electroporation uses an electrical field to disturb the EV membrane, thus, creating pores through which active agents can be loaded into EVs [46]. Electroporation can load nucleic acids [51,52] and drugs [53] into EVs. Isolated EVs are mixed with cargo in an electroporation buffer, which can be either sucrose-based [54], trehalose-based [54], or contain 1.15 mM (pH = 7.2) potassium phosphate, 25 mM potassium chloride, and 21% OptiPrep (Sigma-Aldrich, 14508 St. Louis, MO 68178, United States) [43,51,52,53,55], 272 mM sucrose, 7 mM (pH = 7.40 di-Potassium hydrogen phosphate, and 1 mM magnesium chloride [54]. Depending on cargo type, electroporation is carried out to load cargo into EVs at a voltage and capacitance. Then, EVs are isolated using centrifugation and microfiltration [43,51,52,53,55]. To maximize cargo loading, EV concentration, cargo concentration, electric pulse voltages, and capacitances must be optimized [54]. Lennard et al. [54] proposed the most effective doxorubicin loading method using a doxorubicin–EV ratio of 1 mM:5 × 1011 incubated in a buffer of 400 mM sucrose and electroporated at 950 V and 50 µF.

### 3.6. Sonication

The first step in the sonication method is incubating cargo with EVs at room temperature for 30 min in 1.5 mL tubes. After that, sonication is performed in a water bath sonicator at 35 kHZ for the 30 s and repeated after placing tubes on ice for 1 min [52]. Loading of siRNA, miRNA, and ssDNA was greater than passive loading control (325%, 267%, and 225%, respectively) (Table 3) [52].

## 4. Extracellular Vesicles as Drug Transporters—Practical Examples

Recent findings have pointed out extracellular vesicles (EVs) as potential therapeutic tools to attack HIV infection, given their pivotal role in mediating important cell-to-cell communication mechanisms. EVs may block HIV infectivity, control infection, modulate the immune response, and deliver anti-HIV factors to target cells [59]. For example, research conducted by Elsharkasy et al. [2] showed that cells treated with special vesicles (EVs expressing vesicular stomatitis virus glycoprotein) displayed a reduction in the copy numbers of HIV provirus and the viral protein Nef. Moreover, EVs from other body fluids have also shown anti-HIV effects. EVs isolated from healthy donors’ breast milk have a protective role in vitro. EVs isolated from vaginal fluid could block HIV in vitro at post-entry steps, most likely by halving the reverse transcription and the integration processes [60,61]. This evidence shows the potential application of extracellular vesicles in treating HIV infection as a novel solution to combination antiretroviral therapy (cART).

The therapeutic potential of EVs is also supported by clinical data emerging from cancer. For example, in glioblastoma (GBM) patients, the levels of PD-L1 DNA in serum-and plasma-derived EVs correlated with tumor volume [62]. Khan et al. [63] showed that poloxamer 188-coated NPs could be used for intracranial glioblastoma treatment. Poloxamers are nonionic triblock copolymers composed of a central hydrophobic chain of polypropylene flanked by two hydrophilic polyoxyethylene chains. Clathrin-mediated endocytosis is the primary mechanism involved in the internalization of NPs, depending on the surface characteristics of NPs. After entry to U87 glioma cells, NPs accessed and released doxorubicin (DOX) in lysosomes, and the drug was transported to nuclei for its cytotoxicity. These results were consistent with the potential of poloxamer 188 binding to Apo proteins and facilitating receptor-mediated endocytosis of nanocarriers [63].

Pascucci et al. [57] demonstrated that MSC-derived EVs could package and deliver paclitaxel (PTX), and PTX-containing EVs have a strong anti-proliferative activity on human pancreatic cancer cells. Study carried out by Haney et al., showed the high anticancer efficacy of macrophage-derived EVs loaded with PTX (EV-PTX) and Dox (EV-Dox) in a mouse model of pulmonary metastases [64]. Another study [65] which focused on EVs from red blood cells (RBC-EVs) loaded with doxorubicin or sorafenib (SRF) showed enhanced therapeutic effects on a murine model of orthotopic liver cancer through a mechanism dependent on macrophages—the growth of orthotopic liver cancer was inhibited. RBC-Evs loaded with drug showed no systematic toxicity, whereas routine doses of DOX and SRF showed systemic toxicity at therapeutically effective doses. Furthermore, Zhang et al., showed that drug-loaded RBC-EVs have a simple production process, so they are promising for the treatment of liver diseases [65].

Kim et al. [66] demonstrated that PTX-loaded, macrophage-derived EVs result in more cytotoxicity in P-gp-positive drug-resistant MDCK cells than the free drug alone. Moreover, PTX prodrug and cucurbitacin B-loaded nano micelles caused the inhibition of tumor growth and captured CTCs to suppress cancer metastasis in breast cancer mouse models [67]. Dendritic cell-derived EV (DEX) administration into patients with melanoma and non-small cell lung cancer (NSCLC) shows modest T-cell activation in clinical trials [68,69]. DEX from interferon-γ maturated DCs boosts the anti-tumor response of NK cells and achieves better progression-free survival in advanced unresectable NSCLC patients [70]. However, receiving highly efficient anti-tumor effects in cancer patients is difficult, probably due to the complicated tumor microenvironment. More research is needed to find the possibility of engineering DEX to improve their therapeutic efficacy. In summary, utilizing engineered EVs as drug transporters in therapy is promising.

Utilizing engineered EVs as drug transporters in therapy is promising. The specificity of using exosomes as a drug carrier creates opportunities for treatments of many inflammation-related diseases. Research carried out by Sun et al., showed that incorporation of curcumin into exosomes can increase the solubility, stability, and bioavailability of curcumin [39]. Exosomal curcumin-treated macrophages produced significantly less IL-6 and TNF-α in comparison with curcumin treatment alone. A study investigating the ability of plant exosomes to deliver curcumin to normal and colon cancer tissue is in phrase 1 trial [71].

Research using mesenchymal stromal cell-derived exosomes with KRAS G12D siRNA in treating 28 patients with metastatic pancreas cancer with KrasG12D mutation is in phrase 1 [72]. Another trial using tumor antigen-loaded dendritic cell-derived exosomes on patients with non-small cell lung cancer is in phase 2 [73]. It is need to be highlighted that the production of artificial EVs can overcome challenges related to sterility, mass production, and regulation [13].

Preclinical studies of EVs as drug delivery systems for cardiovascular disease treatment include acute myocardial infarction, myocardial ischemia reperfusion injury, cerebral ischemia, and cardiotoxicity [74,75,76]. Most of these studies showed the potential therapeutic effects of EVs.

EVs can also be potentially used in treatment of neurological diseases, such as Alzheimer’s disease (curcumin as active pharmaceutical ingredient—API) and Parkinson’s disease (Anti-alpha-synuclein shRNA-minicircle as API) [77,78].

Despite these promising results, more insights are needed to unlock EVs; full potential, comprehensively assess the risk–benefit ratio, and to establish final conclusions.

## 5. Conclusions

Despite the difficulties in standardization and high-throughput production processes, a growing body of evidence indicates that using EVs in drug therapy is prospective. The therapeutic potential of EVs is supported by clinical data emerging from the field of cancer, for example, glioblastoma, pancreatic cancer, lung cancer, or breast cancer. EVs as drug delivery systems may be use for cardiovascular and neurological disease treatment. Nevertheless, more research is needed to find the possibility of receiving highly efficient anti-tumor effects in cancer patients.

## Figures and Tables

**Table 1:** Characterization of subtypes of EVs: exosomes, ectosomes, and apoptotic bodies.

EVs	Size	Orgin and Description	Basic Protein Markers
Exosomes	30–150 nm	An endosomal route forms exosomes through early endosomes, which mature into multivesicular bodies (MVBs). MVB and exosome formation and release are regulated through the endosomal sorting complexes required for the transport (ESCRT) pathway [6].	Tetraspanin family proteins (CD9, CD63, CD81), heat shock proteins (HSP70 and HSP90), proteins involved in sorting and endosomal transport, such as TSG100 and Alix, and sphingolipid ceramides [7,8,9].
Ectosomes/microvesi-cles (MVs)	100–1000 nm	MVs are formed by direct outward budding of the cell’s plasma membrane. The route of MV formation is thought to require cytoskeleton components, such as actin and microtubules, molecular motors (kinesins and myosins), and fusion machinery (SNAREs and tethering factors) [5,10]	Selectins, integrins, CD40L, phosphatidyl serine, cell-specific markers [11].
Apoptotic bodies (ApoBDs)	50 nm–5 μm	They are formed as a result of cell fragmentation during the process of programmed death (apoptosis) [5].	Due to the mechanism of their formation, they are enriched in histone proteins and phosphatidylserine, and may contain DNA fragments and organelles [10].
		Due to increased hydrostatic pressure following cell contraction, the plasma membrane separates from the cytoskeleton to form these bodies [10].	

**Table 2:** Characterization of techniques used for EVs isolation.

Isolation Category	Examples	Description	Advantages	Disadvantages	Ref.
Density-based	Differential ultracentrifugation	EVs are isolated based on size and density. Several centrifugations allow users to discard cells, large vesicles, debris, and precipitate exosomes.	Cheap, requires little technical experience, no sample pretreatment	Low recovery, time-consuming, less efficient when used on bodily fluids	[19,20,21]
	Density gradient centrifugation	EVs are separated based on their density and size in a centrifuge tube filled with a preconstructed density gradient, made with sucrose or iodixanol.	Very effective, especially for separating EVs from bodily fluids	Low recovery, time-consuming	[21,22,23,24]
Size-based techniques	Ultrafiltration	Separation occurs based on EV size—particles larger than the molecular weight cut-off of a membrane filter are retained and smaller ones pass through into the filtrate.	Less time-consuming than ultracentrifugation, no special instrumentation needed	Loss of EVs on filter unit, particle deformation and lysis of exosomes	[23,25]
	Exosome isolation kit	Sample passed through syringe with two membranes with a 200 nm filter at the top and 20 nm filter at the bottom. Sample pretreated with low-speed centrifugation and proteinase K.	Commercially available, no loss of EVs thanks to pretreatment	Sample pretreatment	[26]
	Size exclusion chromatography	Sample is passed through a column packed with porous stationary phase.	Precise vesicle separation, preserves vesicle structure, integrity, and biological activity	Time-consuming, not easily scalable, cannot be used for high throughput applications	[27,28,29]
Affinity-based techniques	Enzyme-linked immunosorbent assay (ELISA)	Isolation occurs by binding an antigen on EVs with immobilized antibody placed on the surface of a microplate.	Isolation of specific subset of EVs	Not used in clinical settings	[23,30]
	Magneto-immunoprecipitation	Antibody against the antigen on EVs is attached to the surface of streptavidin-coated magnetic beads; then, beads are incubated with EV sample.	Isolation of specific subset of EVs, Quicker than other methods, more pure isolation, no advanced instrumentation needed, larger sample size than ELISA, better at preserving biological activity of exosomal proteins	High reagent cost, low capacity, and low yields	[23,31]
Exosome Precipitation	Polyethylene glycol (PEG) precipitation	Polyethylene glycol, a water-excluding polymer, is added to the sample, which causes other particles, including EVs, to precipitate. To reduce contamination, such as extracellular proteins or protein aggregates, a pretreatment, i.e., ultracentrifugation is needed.	Quick, no expensive equipment needed, no experience needed, variety of starting volumes (100 µL to several mLs)	Lack of selectivity, pretreatment needed	[21,23,25]
	Lectin-induced agglutination	Lectins, added to EVs sample, bind to carbohydrates on the surface of EVs which causes them to precipitate out the of solution. Similarly to PEG precipitation, a pretreatment is needed to avoid contamination.	Quick, no expensive equipment needed, no experience needed	Pretreatment needed	[32]
Microfluidic-based Isolation	Acoustic nanofilter	The EV sample is injected into a chamber and exposed to ultrasound waves that cause particles in the sample to migrate towards the pressure node.	Quick, Low starting volume, Minimal expertise and training	In development stages	[33]
	Immuno-based microfluidic isolation	EVs are separated from the sample due to binding with antibodies immobilized on a microfluidic chip against antigens on the surface of EVs. No pretreatment is needed.	Quick, low starting volume, allows users to isolate EVs from bodily fluids, minimal expertise and training		[34]

**Table 3:** Characterization of techniques applicable for loading EVs.

Method	EV Type	Cargo	Conditions	Ref.
Electro- poration	Ezosome, microvesicle	siRNA, miRNA, dsDNA	Samples incubated at room temperature for 15 min, then centrifuged at 5000× *g* at 4 °C for 5 min	[52]
	Exosome	Doxo-rubicin	Cargo mixed with 200 µL of electroporation buffer at 4 °C, then electroporated at 350 V and 150 µF in 0.4 cm electroporation cuvettes, incubated at 37 °C for 30 min, and centrifuged at 120,000× *g* for 90 min	[53]
	Exosome	miRNA	Electroporation at 0.5 kV 5× with 10-ms pulses, then centrifuged at 100,000× *g* for 120 min at 4 °C	[43]
Sonication	Exosome	siRNA, miRNA, and dsDNA	Cargo incubated with 100 µg of exosomes at room temperature for 30 min, then sonicated in a water bath sonicator at 35 kHZ for 30 s	[51]
Transfection of exosome-producing cell and genetically modified cell	Exosome	miR-9	Cells centrifuged at 100,000× *g* overnight, then stored at 4 °C for 24 h. They were then washed twice with PBS and cultured with exosome-depleted media for 82 h. Then, the media were centrifuged at 2000× *g* for 20 min. After that, the supernatant was centrifuged at 10,000× *g* for 30 min, then again at 100,000× *g* for 80 min twice	[56]
	Exosome	Paclitaxel	Cells incubated for 24 h, then centrifuged at 2500 × *g* for 15 min. Supernatant centrifuged at 16,500× *g* for 20 min, then again at 110,000× *g* for 70 min	[57]
	Exosome	HGH siRNA	Cells incubated for 24 h. Transfection using lipofectamine 2000	[58]
Chemical-based transfection	Exosome	miRNA	miRNAs are mixed with exosomes in a PBS buffer and CaCl_2_ (concentration of 0.1 M). Next, the sample is cooled in ice, then rapidly heated at 42 °C and subsequently cooled again in ice	[43]
	Exosome	siRNA	siRNA was mixed with lipofectamine and incubated for 10 min at room temperature. Next exosome suspension was added and incubated for 30 min.	[59]
	Exosome	siRNA	siRNA was incubated in PBS solution with HiPerFect for 10 min at room temperature. The excess siRNA was purified using latex beads (Sigma-Aldrich, 14508 St. Louis, MO 68178, United States)	[45]
Incubation with membrane permeabilizers or passive	Exosome	Curcumin	Incubation 5 min at 22 °C (EVs, curcumin) Centrifugation in a sucrose gradient (8, 30, 46, and 60%) for 1.5 h at 36,000 rpm	[39]
	Exosome Shedding microvesicles	Porphyrins	Incubation for 10 min at room temperature evs, porphyrins, and saponins (concentration of 0.1 mg/mL)	[48]
	Exosome	Qithaferin A; bilberry-derived anthocyanidins; curcumin; paclitaxel; docetaxel	Cargo dissolved in ethanol or 1:1 mixture of ethanol and acetonitrile Incubation at 22 °C, centrifugation 10 min 10,000× *g* and 2 h 135,000× *g*	[47]
Extrusion	Exosome	Porphyrin	A polycarbonate 400 nm pores diameter membrane in a syringe-based hand-held mini-extruder at 42 °C, ×31 times	[48]
	Exosome	Catalase	200 nm-pore diameter membrane Avanti lipids extruder ×10 times	[49]
Freeze and thaw cycles	Exosome	Catalase	3 repeated cycles: 30 min of incubation of the exosomes in PBS buffer with catalase at room temperature, rapid freezing of the samples at −80 °C, and re-thawing	[49]
	Exosome	Peptide	3 repeated cycles: 2 h of incubation of the exosomes in PBS buffer with peptides at room temperature, rapid freezing of the samples at −80 °C, and re-thawing	[50]

## Data Availability

No new data were created or analyzed in this study. Data sharing is not applicable to this article.
